# Socioenvironmental determinants as indicators of plague risk in the central highlands of Madagascar: Experience of Ambositra and Tsiroanomandidy districts

**DOI:** 10.1371/journal.pntd.0011538

**Published:** 2023-09-06

**Authors:** Sitraka Rakotosamimanana, François Taglioni, Masiarivony Ravaoarimanga, Minoarisoa Esther Rajerison, Fanjasoa Rakotomanana

**Affiliations:** 1 Institut Pasteur de Madagascar, Antananarivo, Madagascar; 2 Université de La Réunion, France; 3 UMR Prodig, Paris, France; University of Texas Medical Branch at Galveston, UNITED STATES

## Abstract

**Background:**

Human plague cases are reported annually in the central highland regions of Madagascar, where the disease is endemic. The socioenvironmental characteristics and lifestyles of the populations of the central highland localities could be linked to this endemicity. The aim of this study was to determine socioenvironmental determinants that may be associated with plague risk and explain this variation in epidemiological contexts.

**Methods:**

The current study was based on the distribution of plague cases between 2006 and 2015 that occurred in localities of districts positioned in the central highlands. Household surveys were performed from June to August 2017 using a questionnaire and direct observations on the socioenvironmental aspects of households in selected localities. Bivariate and multivariate analyses were performed to highlight the socioenvironmental parameters associated with plague risk in both districts.

**Results:**

A total of 503 households were surveyed, of which 54.9% (276/503) were in Ambositra and 45.1% (227/503) were in Tsiroanomandidy. Multivariate analyses showed that thatched roofs [adjusted odds ratio (AOR): 2.63; 95% confidence interval (95% CI): 1.78–3.88] and ground floor houses [AOR: 2.11; 95% CI: 1.3–3.45-] were significantly associated with the vulnerability of a household to plague risk *(p value<0*.*05*).

**Conclusions:**

Plague risk in two districts of the Malagasy central highlands is associated with human socioenvironmental characteristics. Socioenvironmental characteristics are parameters expressing spatial heterogeneity through the difference in epidemiological expression of the plague in Ambositra and Tsiroanomandidy. These characteristics could be used as indicators of vulnerability to plague risk in plague-endemic areas.

## Author summary

Plague is an animal disease that can affect humans. The disease involves three main actors: the bacillus, the flea, and the rodent. Madagascar is the country with the most human cases of plague in the world. Each year, cases of plague are reported there. This is due to several factors, including the climate and altitude, which are favorable to rodents and fleas. Factors that may contribute to the occurrence of human cases of plague are often those that facilitate contact between human rodents and fleas. The lifestyles of people, their types of housing, and their environment may be some of the factors that facilitate this contact. In plague-endemic areas of Madagascar, households with thatch-roofed, ground floor house types are more likely to have human plague cases compared to households with other characteristics. Households with these characteristics may provide shelter and protection for rodents and are more attractive to rodents, promoting flea-rodent-human contact. This study provides insights into the mechanism of human plague occurrence in Madagascar and other countries.

## Introduction

Plague is a zoonotic disease that mainly affects animal populations and involves three main actors in its transmission cycle: the bacillus *Yersinia pestis*; the flea vector; and the rodent host reservoir. Humans are accidental hosts of the disease [1,2]. Currently, the human plague continues to affect a few regions of the world [3]. From 2013 to 2018, according to the World Health Organization (WHO), 2886 human plague cases were reported worldwide, including 504 deaths [3]. While the American and Asian continents recorded plague cases during this period, most cases were reported on the African continent. Four countries, namely, Uganda, the Democratic Republic of Congo, the United Republic of Tanzania (URT), and Madagascar, reported approximately 97% (2791/2886 cases) of the human cases of plague from 2013 to 2018 [3]. Plague may circulate within animal populations, including rodent populations and vector fleas, with natural plague focus spreading over several worldwide regions. Population dynamics of vectors and reservoirs are correlated with climate and environmental change. These climate changes and environmental upheavals influence the behavior of rodent populations. The movement of murine populations and their migration to anthropogenic habitats increase the risk of contact between humans and rats [4]. Shelter and food sources provided by human houses and their environment increase this risk of contact [4].

Plague was introduced into Madagascar in 1898 in the port city of Toamasina [5] on the eastern coast. Then, it gradually spread inland and reached the central highlands [5,6]. Since 1921, above 800 meters in altitude in the Malagasy central highlands, plague has been endemic [6,7]. However, sporadic plague cases may occur in non-endemic areas. Even if plague is one of the neglected diseases, it remains a public health problem in Madagascar. Approximately 400 human cases and deaths due to plague are reported annually, particularly in rural areas in the central highlands [6,8,9]. However, unusual plague epidemics occurred in the urban areas. The pulmonary plague outbreak mostly affected the urban cities of Antananarivo and Toamasina in 2017.

In Madagascar, the occurrence of human cases of plague could be linked to climatic conditions and ecological factors [10–13]. The persistence of the disease, its circulation, and the re-emergence of human cases of plague in Madagascar may suggest a link between these cases and the anthropogenic habitats, the spatial practices, and the human actions. Indeed, in addition to the biological factors linked to fleas and rats, human actions on the environment, the spatial and social practices of populations within the central highlands, and the organization of human societies in these areas could have an impact on the occurrence of human plague. These actions have an impact on the ecology of the rat and therefore on the pathogenic complex [14] formed by the main actors in the plague transmission cycle. The practice of *tavy*, or burning cultivation, is an example of the influence of human practices on the occurrence of human plague [4,10]. This practice causes field rats to migrate to human villages for shelter and food. These spatial practices may also explain the differences in epidemiological expressions of plague in different localities. Indeed, the epidemiological contexts are different according to the areas of the central highlands where human common bubonic plague occurs. Periodically, in some areas, cases of plague appear alternated by a (long) period of silence, years without human cases. In other outbreak areas, plague cases occur annually. It has previously been observed that landscapes shaped by people, including human habitats, could promote contact between people and rodents, especially in rural areas of the central highlands [15]. A study performed in a district in the central highlands of Madagascar, other than the ones we studied, showed that low-rise house types with thatched roofs and mud walls were at increased risk for human plague [15].

In fact, a study of people’s Knowledge, Attitudes, and Practices (KAP) in relation to plague was performed in parallel with the present study in the two study sites [16] had as its main objective to assess the links between socio-spatial determinants and people’s levels of KAP in relation to plague in the central highlands. The study highlighted the influence of population KAP levels on plague endemicity in the districts of Ambositra and Tsiroanomandidy. The KAP levels of the populations in these districts are correlated with their lifestyles, behaviors, and social categories, the results of the present study and those of the KAP study could be complementary.

The purposes of the present study were: i) to assess the socio-environmental characteristics of households in two districts of the Malagasy central highlands; and ii) to highlight the socio-environmental parameters associated with plague risk in these two localities.

## Methods

### Ethics statement

The protocol of this study received the authorization of the Ethics Committee at the Ministry of Health of the Republic of Madagascar (N#50 MINSAN/CE on April 26 2016). Study participants were provided with information pertaining to the conduct of the study, the right to refuse to participate, and the guarantees of confidentiality of their personal information. We obtained individual written consents from the study participants. Formal consent was obtained from the parent/guardian for individual under 18 years.

### Study sites

**Fig 1:**
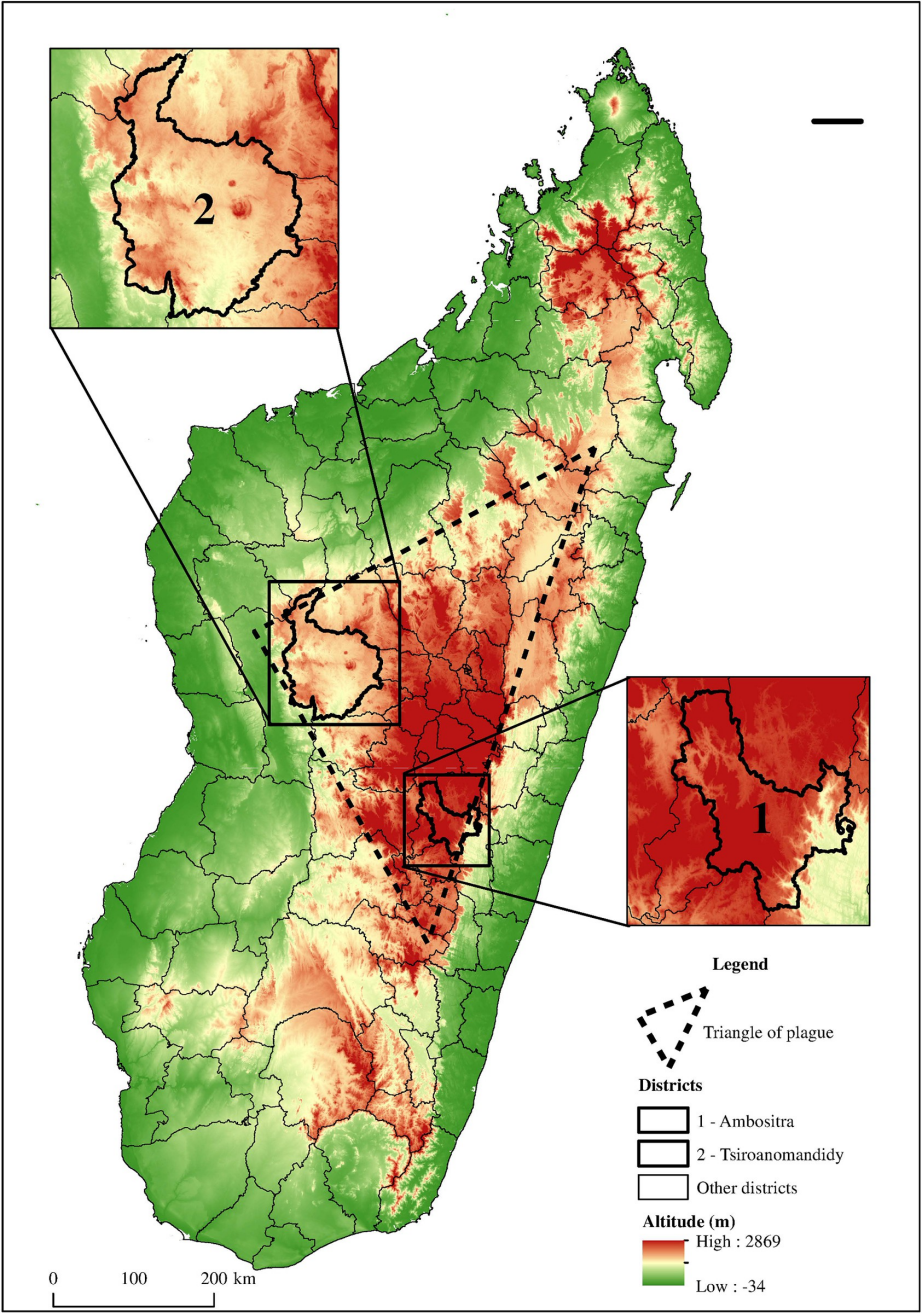
Map of districts locations, the “Triangle of plague”, and altitudes of areas. Performed by M. Ravaoarimanga using Arc GIS 10. 8 software. Shapefile source for administrative limits: BNGRC (National Disaster Management Office), polygons cleaned and merged by UNOCHA (United Nations Office for the Coordination of Humanitarian Affairs, https://www.unocha.org/) in December 2017:—All maps are in the public domain, (https://data.humdata.org/dataset/cod-ab-mdg). Raster source for altitude: U.S. Geological Survey (USGS)—All maps are in the public domain. (https://earthexplorer.usgs.gov/).

Two sites located in plague-endemic areas of the central highlands [Fig 1] were included in the study: (i) the district of Tsiroanomandidy, an active endemic area with an annual occurrence of human plague cases between 2006 and 2015 according to the database of the *Laboratoire Central Peste* (LCP) at the Institut Pasteur de Madagascar (IPM), and (ii) the district of Ambositra, an active endemic area. Between 2006 and 2015, periods of silence were detected in this district, according to the IPM database.

### Study design

This study is a prospective and comparative descriptive analysis based on household investigations conducted in two plague-endemic districts of the Malagasy central highlands. The study was performed in two steps: (i) the selection of municipalities to be investigated in the districts of Tsiroanomandidy and Ambositra and (ii) the conduct of household surveys in the field to document the socioenvironmental characteristics of households that may be linked to human plague occurrence.

### Selection of municipalities to be investigated

The database of the LCP was used to determine the distribution of plague cases in the study sites between 2006 and 2015. This database contains all the information relating to human plague cases reported or observed in health facilities located at different administrative scales in Madagascar: date of registration of the individual, categories of plague cases of the individual (suspected, probable or confirmed), clinical forms of plague contracted (bubonic, pulmonary, or other), and the status of the patient (living or dead). And socio-demographic informations of individual registered in this database: marital status; last name; first name; sex; age; and address of individuals registered in this database. All individuals residing in study districts that have been categorized as probable and confirmed cases of all forms of plague, living or dead, were included in the study. This distribution was the basis for the choice of municipalities to be investigated in the second step of the study. Two types of municipalities were categorized: (i) municipalities that reported at least one probable or confirmed case of plague, and (ii) municipalities that did not report any cases of plague. The common cases and controls per district to be investigated were selected separately. Therefore, all localities had the chance to be chosen. Locations that were not geographically accessible were not selected for investigation.

In Madagascar, a municipality is an administrative delimitation component of the district and is composed of several fokontany. The fokontany is the smallest official administrative unit.

In the field, the *fokontany* of each municipality was categorized into two subcategories:

The *fokontany* with plague cases between the study period and the *fokontany* without cases for the same period.

Data on the distribution of plague cases in the two districts were published in a previous scientific paper [17].

**Fig 2:**
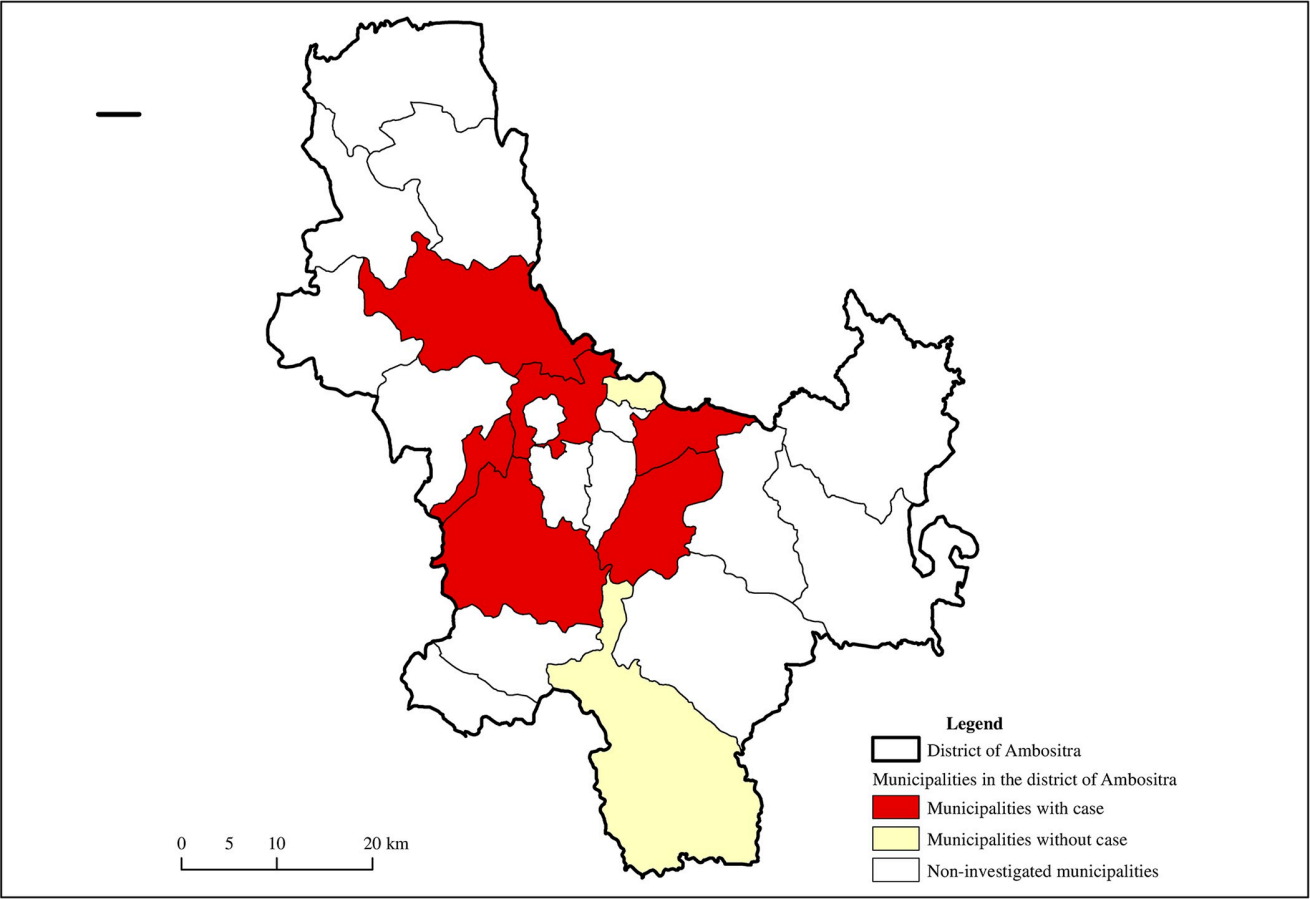
Locations and status of investigated municipalities in the Ambositra district. Performed by M. Ravaoarimanga using ArcGIS 10.8 software. Shapefile source for administrative limits: BNGRC (National Disaster Management Office), polygons cleaned and merged by UNOCHA (United Nations Office for the Coordination of Humanitarian Affairs/ https://www.unocha.org/) in December 2017: all maps are in the public domain and open to the public https://data.humdata.org/dataset/cod-ab-mdg.

**Fig 3:**
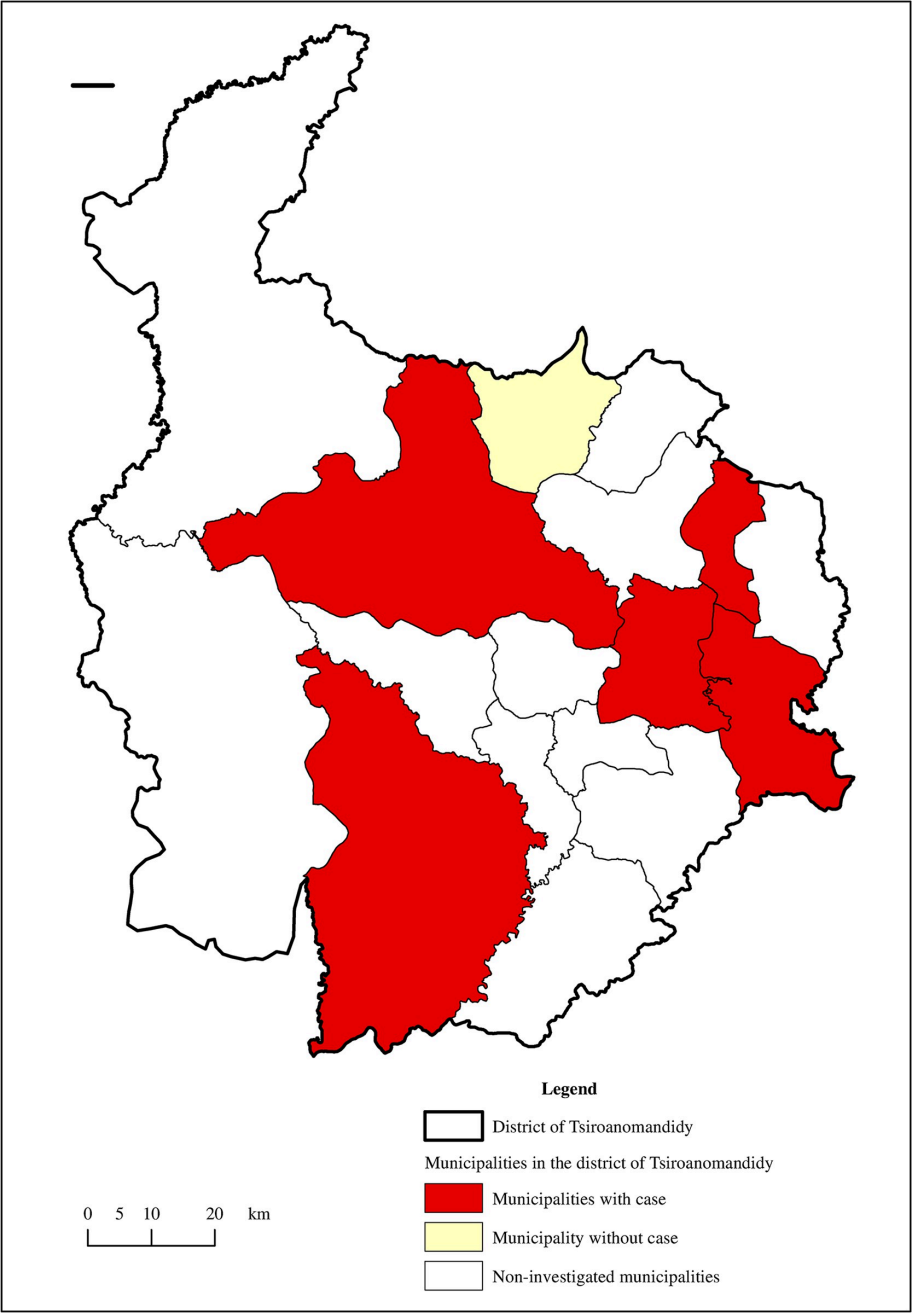
Locations and status of investigated municipalities in the Tsiroanomandidy district. Performed by M. Ravaoarimanga using ArcGIS 10. 8 software. Shapefile source for administrative limits: BNGRC (National Disaster Management Office), polygons cleaned and merged by UNOCHA (United Nations Office for the Coordination of Humanitarian Affairs/ https://www.unocha.org/) in December 2017; all maps are in the public domain and open to the public, https://data.humdata.org/dataset/cod-ab-mdg.

For the Ambositra district, a total of 7 municipalities [Fig 2] were investigated, including 5 municipalities with cases and 2 municipalities without cases between the selected periods. The surveys and observations were performed in *10 fokontany*. For the district of Tsiroanomandidy [Fig 3], 7 municipalities, of which 6 were case municipalities and one was a municipality without cases, were investigated. Surveys and observations were conducted in 10 *fokontany*. A total of 503 households were surveyed.

### Household surveys and field observations

A team of investigators from the Institut Pasteur de Madagascar performed household surveys from June to August 2017 in the districts of Ambositra and Tsiroanomandidy to assess their socioenvironmental characteristics. In addition to the questionnaire administered to the household representatives, observations by investigators on the characteristics of the household environment were performed. The purpose of the observations was to describe the visible features of the homes surveyed. These observations concerned i) the house features: type of roof; type of wall, and one-floor or ground floor house [S1 Fig]; and ii) the socioenvironmental features of the houses: presence of fences < 10 meters around buildings; presence of bushes < 10 meters around buildings; and presence of sisal hedge).

The questionnaire was coupled with an individual survey of family members. The questionnaire focused on behaviors and perceptions, including a Knowledge, Attitudes, and Practices (KAP) study [16] on plague in the population of both districts. In the KAP study, an individual survey about plague was conducted in the two districts. The KAP study in relation to population-based plague was conducted simultaneously with this socio-environmental study. However, the mode of selection of participants was the same for both studies. We estimated that 20 households should be visited in each fokontany investigated, with a maximum of two individuals interviewed per household, in order to have 30 individuals investigated per fokontany. The respondent representing the household was drawn at random among the members of the household over 16 years of age.

All individuals residing in the fokontany drawn at random from the two study districts, who agreed to participate in the survey and signed an informed consent form were also included. The questionnaire was administered in Malagasy. Each interview was preceded by an information session on the study.

### Data processing and statistical analysis

The characteristics of households and their socioenvironmental components were summarized by descriptive analyses using Microsoft Excel and Stata 13 software. Qualitative variables are represented as frequencies or proportions. Either a chi-square test or Fisher’s exact test, if appropriate, was used to establish the association between categorical variables.

Multivariate analysis was performed to determine the associations between the different household characteristics and the epidemiological status of a locality or *fokontany* (absence or occurrence of plague cases between 2006 and 2015). These associations were analyzed on the basis of a stepwise binomial logistic regression. Stepwise binomial logistic regression was used to select variables that may explain vulnerability to plague risk (1 = yes, 0 = no) for inclusion in the final model and to remove those that are statistically non-significant.

All independent variables with a *p value ≤ 0*.*25* were retained in the bivariate analysis and then included in the logistic regression model to determine the associations between the variables. A *p value < 0*.*05* was considered statistically significant in the final model.

## Results

### Household characteristics

Of the 503 households, the average size was 5.3 individuals per household. At least one household was composed of only one-individual and the largest household was composed of 19 individuals. On average, a household consisted of three adults and two children less than 15 years of age.

For all households surveyed, a total of 87.7% (441/503) raised farm animals (cattle—pigs), of which 48.9% (246/503) were located in the Ambositra district and 38.7% (195/503) were located in the Tsiroanomandidy district. Approximately 39% (198/503) of households reported having a dog and/or cat, including 24.2% (122/503) in Ambositra and approximately 15% (76/503) in Tsiroanomandidy. The possession of a cat or dog was significantly associated with the epidemiological status of a fokontany (*p value* = *0*.*030*) (Table 1). Households with pets in Ambositra district were more numerous than those in Tsiroanomandidy [Ambositra: 44% (122/276) *versus* Tsiroanomandidy: 33% (76/227)] (*p value = 0*.*014)*.

**Table 1:** Features of investigated and observed households with bivariate analysis results according to family epidemiological status and districts.

Variables	*Fokontany* status					Total n (%)	*p value*	
	With cases	n	Total	No case	n	Total		
	**Districts**							
	Ambositra	Tsi/didy	n (%)	Ambositra	Tsi/didy	n (%)		
**Household features**								
**Size**								*0*.389*
1 to 3	29	51	80 (28.7)	46	6	52 (23.2)	132 (26.2)	
4 to 6	40	87	127 (45.5)	100	17	117 (52.5)	244 (48.5)	
7 to 9	7	50	57 (20.4)	36	5	41 (18.3)	98 (19.5)	
10 or more	4	11	15 (5.4)	14	0	14 (6.3)	29 (5.8)	
**Animal breeding**								*0*.126*
No	13	27	40 (14.3)	17	5	22 (9.8)	62 (12.3)	
Yes	67	172	239 (85.7)	179	23	202 (90.2)	441 (87.7)	
**Dog/cat ownership**								*0*.030*
No	50	131	181 (64.9)	104	20	124 (55.4)	305 (60.6)	
Yes	30	68	98 (35.1)	92	8	100 (44.6)	198 (39.4)	
**House features**								
**Roof type**								*<0*.001**
Tile	36	52	88 (31.5)	109	3	112 (50)	200 (39.8)	
Thatch	37	145	182 (65.2)	56	25	81 (36.2)	263 (52.3)	
Sheet metal	6	2	8 (2.9)	23	0	23 (10.3)	31 (6.2)	
Other	1	0	1 (0.4)	8	0	8 (3.6)	9 (1.8)	
**Wall type**								*0*.001**
Brick	34	81	115 (41.2)	123	7	130 (58)	245 (48.7)	
Cob	43	112	155 (55.6)	69	21	90 (40.2)	245 (48.7)	
Wooden	0	2	2 (0.7)	2	0	2 (0.9)	4 (0.8)	
Other	3	4	7 (2.5)	2	0	2 (0.9)	9 (1.8)	
**At least one floor house**								*<0*.001*
No	0	81	81 (29)	16	16	32 (14.3)	113 (22.5)	
Yes	80	118	198 (71)	180	12	192 (85.7)	390 (77.5)	
**Stock of farm products inside houses**								*0*.51*
No	15	40	55 (19.7)	38	1	39 (17.4)	94 (18.7)	
Yes	65	159	224 (80.3)	158	27	185 (82.6)	409 (81.3)	
**Socioenvironmental features**								
**Fenced houses**								*0*.463*
No	72	164	236 (84.6)	160	24	184 (82.1)	(83.5)	
Yes	8	35	43 (15.4)	36	4	40 (17.9)	83 (16.5)	
**Sisal hedge**								*0*.217*
No	70	189	259 (92.8)	174	27	201 (89.7)	460 (83.5)	
Yes	10	10	20 (7.2)	22	1	23 (10.3)	43 (16.5)	
**Bushes around 10 meters**								*0*.124*
No	51	165	216 (77.4)	143	17	160 (71.4)	376 (74.8)	
Yes	29	34	63 (22.6)	53	11	64 (28.6)	127 (25.2)	
**Rubbish/trash around 10 meters**								*0*.873*
No	7	18	25 (9)	11	10	21 (9.4)	46 (9.1)	
Yes	73	181	254 (91)	185	18	203 (90.6)	457 (90.9)	

* Bivariate analysis by chi-square test

** Bivariate analysis by Fisher’s exact test

Approximately 81.3% (409/503) of households reported storing agricultural products in their homes, of which 44.33% (223/503) are Ambositra district households and more than 37% (186/503) are Tsiroanomandidy district households. Approximately 91% (457/503) of households reported having a garbage pit or a place where garbage is dumped near their home, and 16.5% (83/503) of homes are reportedly fenced.

### Houses features

For housing construction materials, the type of roof, the type of wall, and ground floor houses were significantly associated with the epidemiological status of a fokontany, with a *p value< = 0*.*001* (Table 1).

The types of walls involved: The brick used in the central highlands is a red laterite clay brick made by hand. Cob walls are built with clay soil mixed with chopped straw. Wooden or brick walls are harder than the more fragile cob walls.

With regard to the types of walls of the buildings, for all the studied districts, in total, almost 49% (245/503) had brick walls. The proportion of houses with a cob wall was also 49% (245/503). On the other hand, 0.8% of houses in both districts had wooden walls (4/503). Houses with brick walls were more represented in Ambositra district [57% (157/276)] than in Tsiroanomandidy district [39% (88/227)], and houses with a cob wall were more represented in Tsiroanomandidy district [58% (133/227)] than in Ambositra district [40% (112/276)]. This difference in proportions in relation to house wall types was statistically significant between the two districts (*p value 0*.*001*).

For the roof types of houses in both districts, approximately 52% (263/503) of the houses had a thatched roof, approximately 40% had a tiled roof, and approximately 6% (31/503) had a tin roof. Thatched houses accounted for 75% (170/227) of the houses in Tsiroanomandidy district and 34% (93/276) of the houses in Ambositra. Tile-roofed houses accounted for 52% (145/276) of the houses in Ambositra and nearly 24% (55/227) of the houses in Tsiroanomandidy. For tin roofs, Ambositra district had a proportion of almost 10% (29/227), while Tsiroanomandidy had a proportion of approximately 1% (2/227). Roof types were significantly different for the two districts *(p value<0*.*001*).

Almost 77% (390/503) were houses with at least one floor. Houses with at least one floor were significantly different between the two districts [Ambositra: 94% (260/276) *versus* Tsiroanomandidy: 57.2% (130/227)] (*p value<0*.*001*).

### Socioenvironmental features

The presence of bins or rubbish areas around houses (<10 meters) concerned 81% (409/503) of houses in both districts. The difference was statistically significant between the two districts [Ambositra: 51% (258/503) *versus* Tsiroanomandidy: 39% (199/503)] (*p value = 0*.*004)*.

The presence of sisal hedges around dwellings (< 10 meters) concerned nearly 8.5% (43/503) of all households in both districts. The difference between the two districts was statistically significant, with Ambositra at 11.5% (32/276) and Tsiroanomandidy at 4.8% (11/227) (*p value = 0*.*007*).

The presence of bushes close to the houses (< 10 meters) concerned nearly 25% (127/503) of the houses investigated. There were more brushes close to the houses located in Ambositra [29.7% (82/276)] than in the houses located in Tsiroanomandidy [19.8% (45/227)] (*p value = 0*.*011*).

### Multivariate analysis

Variables with a *p value < = 0*.*25* were integrated into the univariate binomial logistic regression analysis and then eliminated as they progressed until only variables with a significant *p value < 0*.*05* were retained in the final model.

The variables that were kept in the final model after binomial step-by-step logistic regression were the roof, houses with at least one floor variable, and the cat/dog ownership variable *(p value< = 0*.*05*) (Table 2).

**Table 2:** Multivariate analysis by binomial logistic regression on the influence of each socioenvironmental variable on the *fokontany’s* epidemiological status.

Variables	*Fokontany* status		Unadjusted	Adjusted	95% CI	
	With cases (n)	Without cases (n)	OR	OR	Lower	Upper
**Dog/cat ownership**						
Yes	98	100	1	1	-	-
No	181	124	1.48	1.45	0.99	13
**Roof type**						
Tile	88	112	1	1	-	-
Thatch	182	81	2.85	2.63	1.78	3.88
Sheet metal	8	23	0.44	0.51	0.21	1.22
Other	1	8	0.15	0.12	0.01	1.01
**At least one floor house**						
Yes	198	192	1	1	-	-
No	81	32	2.45	2.11	1.3	3.45

According to the results of the multivariate analysis, compared to households with a tiled roof, households with a thatched roof [Adjusted Odds Ratio (AOR): 2.63; 95% confidence interval (95% CI): 1.79–3.89] *(p value<0*.*001*) had an increased risk of vulnerability to plague and an increased likelihood of being positioned in a locality where plague cases were reported during the study period. Compared to a house with a tiled roof, a house with a roof other than thatch or tin had a decreased probability of being exposed to plague risk [AOR 0.12; 95% CI: 0.01–1.01] (*p value = 0*.*051*).

Households that were low-houses, ground floor-houses, had an increased risk of vulnerability to plague and an increased likelihood of being positioned in a locality where plague cases were reported during the study period compared to houses with at least one floor [AOR: 2.11; 95% CI: 1.3–3.45] *(p value = 0*.*003)*.

Households without a pet had an increased probability of being in a *fokontany* case compared to households that did have a pet [AOR: 1.45; 95% CI: 0.99–2.13] *(p value = 0*.*05*).

## Discussion

The socioenvironmental characteristics of households in the two districts may constitute parameters related to the exposure risk of human cases of plague. These districts are located in the central highlands in Madagascar, which are endemic areas of plague. The materials used to build traditional houses, including thatched roofs, may pose a plague exposure risk to household members.

Our study provides additional information on the vulnerability of populations to plague risk in the particular context of the Malagasy central highlands. Although these elements are not new, to our knowledge, no study of this type has been conducted in the two districts studied.

In the central highlands, single floor houses with thatched roofs are more exposed to plague risk than other house types because of the arboreal characteristics of black rats [18]. Indeed, thatched roofs provide a habitat for the rodent reservoir of plague, especially in rural localities of Malagasy plague foci [15,18,19]. Black rats have been shown to be the rodents involved in the persistence of plague in rural areas of the central highlands [15,18]. Another characteristic of central highland dwellings with a probable association with plague risk is the ground floor houses in the dwellings. Low-rise dwellings, which are often ground floor houses, constitute a risk of contact between rodents and household members. Indeed, for security reasons, in rural areas of the central highlands in particular, people are obliged to sleep in the rooms where they store their agricultural products [18]. This could attract rats and increase the risk of contact between rats, their fleas, and the population [18,19]. Thatched houses provide an ideal habitat for these arboreal rodents [18]. These findings corroborate the results of previous studies conducted [15,19] in the Malagasy central highlands.

Another parameter, namely, the presence of domestic animals such as dogs and cats, is also associated with plague risk for households in both central highlands districts. Indeed, households that do not breed domestic animals, such as dogs and cats, seem to have an increased plague risk. The presence of cats or dogs that are natural predators of rats could then be a protective element against rats and, therefore, a protective factor against plague risk. According to Mahlaba *et al*., the combined presence of cats and dogs, which are natural predators of rats, has an impact on the activities of rodents such as *Rattus rattus* around rural homesteads in Tanzania [20].

The households surveyed in the Tsiroanomandidy district have more of these characteristics than those in the Ambositra district. There are more low-rise dwellings with mud walls in the localities investigated in Tsiroanomandidy district than in Ambositra district. These characteristics may have influenced the epidemiological situations between 2006 and 2015 in both districts, assuming that the humanized landscapes changed little between 2006 and the time of the current investigation.

The occurrence of human cases of plague in a locality is due to several combined factors; the determinants of the pathogen complex formed by the actors of the plague cycle are numerous and of different types [14]. In addition to the biological and ecological factors related to the occurrence of plague cases, human behavior, the organization of humanized spaces, and cultural practices may promote plague risk [18,19,21,22]. Indeed, the lifestyles of rural populations, particularly agricultural practices, are associated with plague risk. The storage of agricultural products in houses, the proximity of fields to houses, and the construction materials used in buildings are all factors that promote contact between humans, rodents, and fleas, increasing the risk of human plague cases. Rodents often seek shelter and food to live [19,22–25]. It has been suggested by Appleby that during the plague epidemics in England in the 16th and 17th centuries, rats lived close to human dwellings in their thatched cottages with plaster walls and low-houses [26]. These building materials are broadly similar to most of the dwellings of the rural populations in the two Malagasy central highlands districts studied. It has also been suggested that improvements in housing, including the use of harder, rat-resistant building materials, were one factor in the gradual disappearance of the plague in the 17th century in England [26,27]. The same is true for the one-room, ground floor houses that promote contact between humans and the main plague actors, as suggested by a study conducted in Tanzania [28], which is another rural area where plague is endemic.

### Limitations of the study

A limitation of the study lies in the fact that the investigated households were randomly selected and is not households or dwellings in which known cases of plague have been reported. The study did not take into account information on the presence or infestation of rodents and their fleas in the home and outdoors. Our study did not focus on the risks of exposure to plague outside the home and does not exclude the risk of infection outside the home. In the framework of our study, we did not collect any data or make any observations on the environment of the localities (fields, presence of rivers, water sources, etc.). The fact that some households were not reached and were not included in our study could be a bias for the study in terms of statistical power.

However, the types of houses as well as the households, in the surveyed districts have very similar characteristics in the central highlands, more specifically in the surveyed districts. Investigating the socioenvironmental characteristics and parameters of households where plague cases have been reported would allow the model to be refined and the socioenvironmental characteristics favorable to the occurrence of plague cases to be validated. Setting up rat traps in different types of houses in rural areas would also confirm whether low-rise houses built with cob walls are more likely to be degraded by rats than other types of houses. The results we have obtained were specific to the context of the Malagasy central highlands and may not reflect the epidemiological situations in relation to plague in other areas of the world.

A perspective for the present study would be to determine and confirm the risk of exposure to *Yersinia pestis* outdoors and indoors by coupling the results of our study with analyses of the infection of rats and their fleas with *Yersinia pestis*.

## Conclusions

The variation in the epidemiological expression of human plague in the Ambositra and Tsiroanomandidy districts may be explained by the socioenvironmental characteristics of humanized areas at different administrative scales. Plague risk exposure may be increased in areas of the central highlands where low-rise dwellings are built of cob. Socioenvironmental characteristics may be indicators that can establish whether a locality presents a plague risk. These characteristics may be combined with other factors of different natures and integrated into a predictive model that may constitute an embryonic alert system for the plague and thus contribute to improving disease control in Madagascar. In addition to the socioenvironmental characteristics of plague-risk-exposure associates, knowing the links between contextualized human behaviors and the occurrence of human cases of plague in the central highlands would be an essential element in adapting plague control interventions to suit the specific contexts of human societies in the central highlands of Madagascar. This approach would help to confirm the link between spatial heterogeneity and differences in epidemiological expression of the plague in central highlands in Madagascar and may explain the endemicity of the plague in these regions.

The heterogeneity of anthropogenic habitat (societies, organizations, cultural contexts, etc.) must be taken into account both in regard to understanding the dynamics of a disease or infection in space and in adapting health messages in terms of information, education and communication for the population.

## Supporting information

S1 Fig Type of ground floor house with cob wall and thatched roof in the district of Tsiroanomandidy. Credits: Sitraka Rakotosamimanana, 2016.
(TIF)

S1 Data Supporting data. (XLSX)
