# Supplementary figures and images for: Socioenvironmental determinants as indicators of plague risk in the central highlands of Madagascar: Experience of Ambositra and Tsiroanomandidy districts
Source: PLoS Negl Trop Dis. 2023 Sep 6;17(9):e0011538. doi: 10.1371/journal.pntd.0011538 (PMC10506711; doi:10.1371/journal.pntd.0011538)

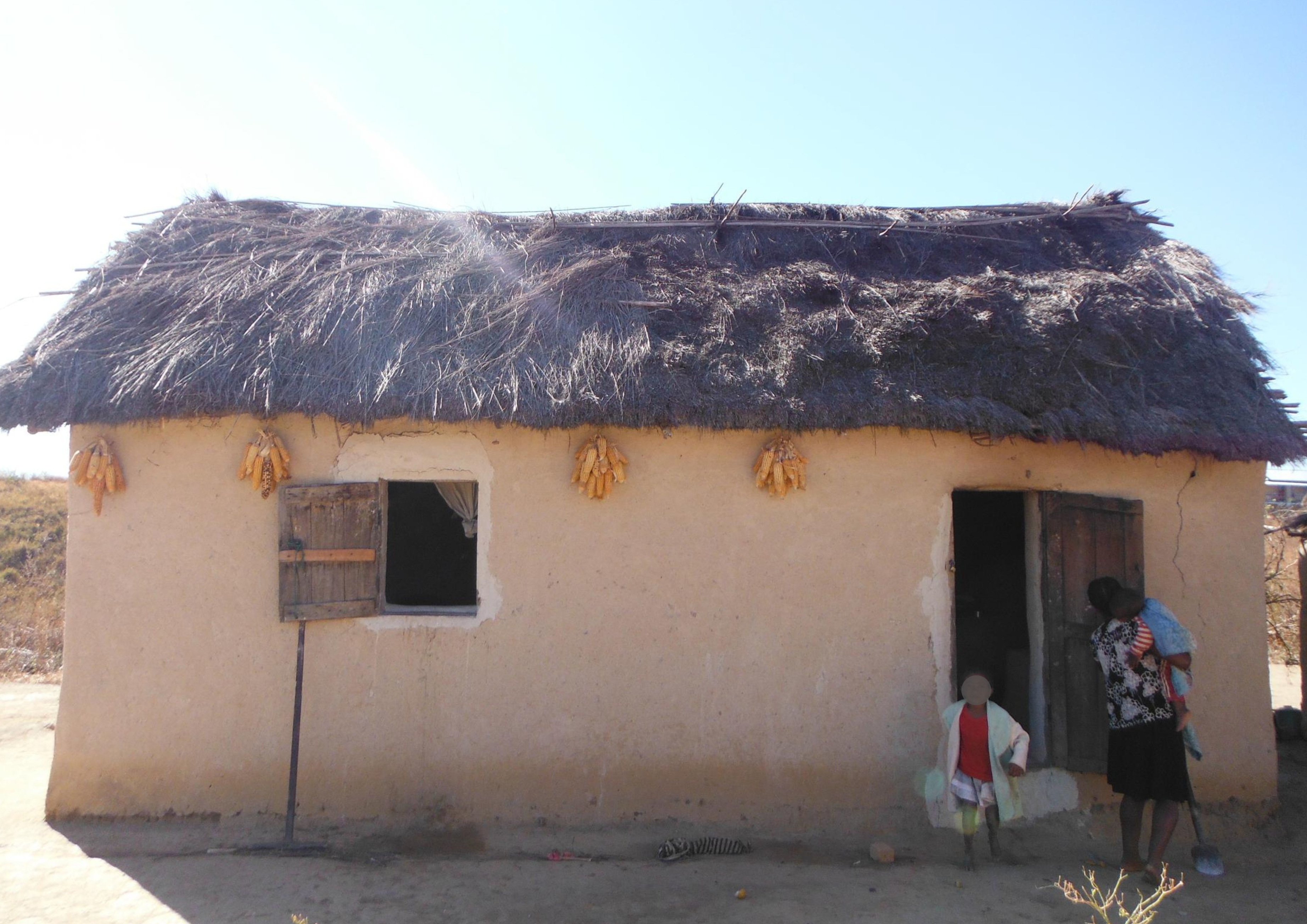

Supplement: S1 Fig — Credits: Sitraka Rakotosamimanana, 2016. (TIF) [file pntd.0011538.s001.tif]
